# The relationship between well‐being and HbA1c in adults with type 1 diabetes: A systematic review

**DOI:** 10.1111/1753-0407.13357

**Published:** 2023-02-16

**Authors:** Aida Pérez‐Fernández, Pablo Fernández‐Berrocal, María José Gutiérrez‐Cobo

**Affiliations:** ^1^ Department of Basic Psychology, Faculty of Psychology University of Málaga Málaga Spain; ^2^ Department of Developmental and Educational Psychology, Faculty of Psychology University of Málaga Málaga Spain

**Keywords:** affective well‐being, cognitive well‐being, HbA1c, systematic review, type 1 diabetes, 认知幸福感, 情感幸福感, 1型糖尿病, 糖化血红蛋白, 系统综述

## Abstract

**Background:**

Diabetes has been associated with psychological problems, which in turn have been related to poorer glycemic control (glycosylated hemoglobin [HbA1c]). On the contrary, psychological well‐being constructs have been associated with superior medical outcomes, including better HbA1c.

**Aim:**

The main objective of this study was to systematically review the existing literature about the relationships between subjective well‐being (SWB) and HbA1c in adults with type 1 diabetes (T1D).

**Methods:**

Comprehensive searches were conducted in PubMed, Scopus, and Medline, time restricted to 2021, for studies examining the link between HbA1c and the cognitive (CWB) and affective (AWB) components of SWB. A total of 16 eligible studies were selected according to the inclusion criteria, of which 15 measured CWB and 1 AWB.

**Results:**

Of the 15 studies included, 11 showed a relationship between CWB and HbA1c, with a higher level of HbA1c being related to poorer CWB. The other four studies did not find any significant association. Finally, the only study examining the relationship between AWB and HbA1c found a marginally association between these variables in the expected direction.

**Conclusion:**

The overall data suggest that CWB is negatively related to HbA1c in this population, but these results are inconclusive. This systematic review offers clinical implications, such as the possible evaluation, prevention, and treatment of the problems associated with diabetes through the study and training of the psychosocial variables that may directly influence SWB. Limitations and future lines of investigation are discussed.

## Abstract 2

**Highlights**

- Subjective well‐being (SWB) is an important variable to consider, given its association with positive outcomes in people with diabetes and the general population.
- The main aim of this study is to systematically review how SWB factors are related to type 1 diabetes management through the glycosylated hemoglobin (HbA1c) index in adults.
- The overall data suggested that the cognitive well‐being is negatively related to HbA1c in this population, but these results are inconclusive.

## 摘要

**背景**:糖尿病常伴有心理问题, 而心理问题又与血糖控制(HbA_1c_)不佳相关。相反, 心理健康与较好的医疗结局(包括较好的HbA_1c_)相关。本研究旨在系统回顾关于成人1型糖尿病(T1D)患者主观幸福感(SWB)和HbA_1c_之间关系的现有文献。

**方法:**检索PubMed,Scopus和Medline, 时间限制至2021年, 以研究HbA_1c_与主观幸福感的认知(CWB)和情感(AWB)成分之间的关系。根据纳入标准共筛选出符合条件的研究16篇, 其中测量CWB 15篇, AWB 1篇。

**结果:**共纳入15项研究, 其中11项研究显示CWB与HbA_1c_相关, HbA_1c_水平越高, CWB越差。其他四项研究未发现任何显著关联。最后, 仅一项研究探讨了AWB和HbA_1c_之间的关系, 结果发现这些变量在预期方向上略有关联。

**结论:**总体数据提示CWB与该人群HbA_1c_呈负相关, 但结果尚不明确。这篇系统综述提供了临床意义, 例如通过对可能直接影响主观幸福感的社会心理变量的研究和培训, 与糖尿病相关的问题可能的评估、预防和治疗。同时讨论了研究的局限性和未来的研究方向。

## 1 INTRODUCTION

Diabetes mellitus is a chronic disease associated with significant morbidity and mortality worldwide.^1^ The global prevalence of diabetes in 2019 is estimated to be 9.3% (463 million people).^2^ More specifically, the prevalence of diabetes mellitus type 1 (T1D) in the world ranges between 0.8 and 4.6/1000 inhabitants.^3^ T1D, which generally begins in childhood, is a disease in which the insulin‐producing beta cells of the pancreas are destroyed.^4^ Therefore, the required treatment consists of daily administrations of exogenous insulin. However, the cause of this type of diabetes is still unknown.^5^

To check how patients control their blood glucose levels, the glycosylated hemoglobin (HbA1c) test is used, which shows the accumulated glycemic history from the previous 2 or 3 months.^6^ The HbA1c test result is given in percentages and has become a reliable indicator for the diagnosis and prognosis of diabetes. According to the American Diabetes Association (ADA), people with T1D are recommended to have an HbA1c level <7%.^7^ In addition, several prospective studies such as the Diabetes Control and Complications Trial (DCCT), The UK prospective Diabetes Study Group (UKPDS), and the Epidemiology of Diabetes Interventions and Complications, have directly linked long‐term diabetic complications with the HbA1c index.^8^, ^9^, ^10^

Problems with maintaining adequate glucose levels make diabetes a highly stressful disease. Therefore, several studies have investigated the relationship between T1D and psychological problems.^11^ Specifically, diabetes has been linked to depression,^12^ anxiety,^13^, ^14^ and stress.^15^ Furthermore, it has been found that these psychological problems are closely related to HbA1c, so the higher the levels of depression, anxiety, and stress, the higher the HbA1c level, implying poorer glycemic control.^11^, ^13^, ^15^, ^16^ In addition to depression, anxiety, and stress, T1D control could affect the subjective well‐being (SWB) of these patients.^17^, ^18^

The concept of SWB described by Diener^19^ refers to how people think and feel about their lives. According to the initial formulation of this author, the SWB was made up of three elements: satisfaction with life, positive affect, and negative affect. In turn, this concept can be divided into two components^19^, ^20^: cognitive well‐being (CWB) and affective well‐being (AWB). CWB refers to the cognitive evaluation of a person's life satisfaction in general and specific life domains (eg, health satisfaction, job satisfaction).^21^ On the other hand, AWB represents both the pleasant and unpleasant affect that a person can experience. This concept, in turn, can be divided into mood and emotions^22^ that act as a monitoring system in the progress and achievement of people's goals and efforts.^23^ AWB and CWB differ in their stability and variability^24^ and are related to other variables.^25^, ^26^ Therefore, it seems logical that various external life events could have a differential impact on an individual's AWB or CWB.^22^

SWB is a crucial variable to consider, given its association with positive outcomes in people with diabetes and the general population. For instance, relevant concepts such as social support and self‐care behaviors are positively related to CWB.^27^, ^28^ In addition, indicators of AWB, such as positive affect, self‐efficacy, optimism, and gratitude, have been associated with good health outcomes in various medical conditions.^29^, ^30^ For example, a systematic review has shown that people with T1D and depressive symptoms also had lower CWB,^31^ and similar results have also been found with anxiety.^13^

Considering the previous literature, the main aim of this study is to systematically review how SWB factors are related to T1D management through the HbA1c index in adults. We hypothesize that higher levels of CWB and AWB will be negatively related to diabetes control, as indicated by favorable (lower) HbA1c levels. Conversely, we expect lower levels of SWB will be associated with poorer HbA1c.

## 2 METHOD

### 2.1 Literature search

PubMed, Scopus, and Medline databases were searched exhaustively, time restricted to 2021, for studies examining the link between T1D and SWB. Searches were conducted using the following keywords in English: “type 1 diabetes” and “HbA1c” combined with “quality of life,” “positive affect,” “negative affect,” “life satisfaction,” “happiness,” and “psychological well‐being” as terms in the title or abstract. Searches were undertaken between September and October 2022. The review was previously registered on the International Prospective Register of Systematic Reviews (PROSPERO) platform with the following ID: CRD42021287996.

### 2.2 Inclusion criteria

To be included in the review, papers had to meet the following requirements: (a) report empirical research providing data on the relationship or predictive capacity of SWB for glycemic control, measured through the glycated hemoglobin index (HbA1c); (b) adult sample with a diagnosis of T1D; (c) use of a valid and reliable cognitive and affective well‐being scale or subscale; (d) of any ethnicity and gender; (e) written in Spanish or English; and (f) cross‐sectional and longitudinal. In addition, we excluded letters, theses, comments, editorials, book chapters on previously published studies, protocol interventions, qualitative studies, articles that do not distinguish between T1D or type 2 (T2D), child or adolescent samples, articles on T2D, and non‐English or non‐Spanish language publications.

### 2.3 Data extraction

**FIGURE 1:**
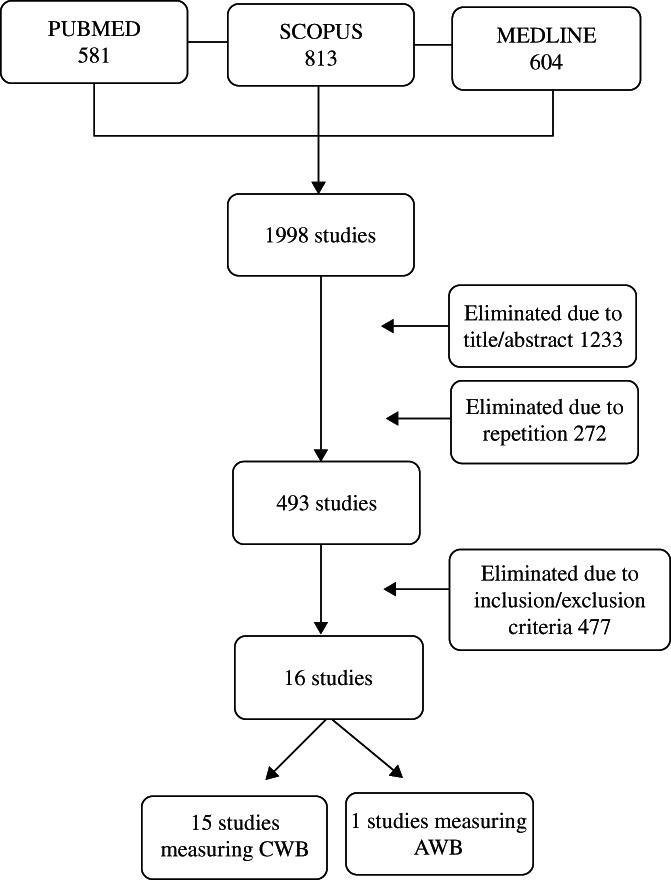
PRISMA flow‐diagram for determining the articles included in this study AWB, affective well‐being; CWB, cognitive well‐being; PRISMA, Preferred Reporting Items for Systematic Reviews and Meta‐Analyses

The initial database search identified 1998 potentially eligible studies: 581 from PubMed, 813 from Scopus, and 604 from Medline. After two reviewers independently assessed the titles and abstracts of all identified reports and duplicates, 1505 studies were eliminated. Only 493 were selected for full‐text review based on the specified inclusion/exclusion criteria. After this, 16 studies were finally included. These articles were divided into two sections: those measuring CWB (15 studies) and those measuring AWB (1 study). Disagreements were resolved by consensus with the lead reviewer. The process of searching and selecting articles is shown in Figure 1. To analyze the quality of the studies, we have included a quality assessment table using the Mixed Methods Appraisal Tool^32^ (see Appendix A, Table A1).

## 3 RESULT

We found 16 studies that linked SWB to HbA1c in adults. More specifically, 15 measured CWB, and 1 measured AWB.

### 3.1 CWB and HbA1c

This section describes the studies that measured CWB with nine different questionnaires (see Appendix B) over the 15 selected articles. A total of 10 articles found associations between the two variables, and four did not find any relationship. We have divided the results into cross‐sectional (11 studies, see Table 1) and longitudinal (4 studies, see Table 2).

**TABLE 1:** Cross‐sectional studies analyzing the relationship between CWB and HbA1c

Study	Sample	Instruments	Outcome measures	Results
Weinger and Jacobson^33^ USA	55 patients with T1D. Mean age 34 ± 8 years. Female, *n* = 31 (56.36%)	DQOL	HbA1c	A higher CWB measured through the satisfaction subscale was associated with a lower level of HbA1c (*r* = −0.30, *p* = .03). This negative relationship was also found between the changes in the same CWB subscale and the changes in HbA1c through the intervention (*r* = −0.34, *p =* .01).
Reddy et al^34^ UK	57 patients with T1D. Mean age = 31.4 ± 11.6 years. Female, *n* = 49 (85.9%)	DQOL	HbA1c	HbA1c did not correlate with CWB in the overall study population. On the other hand, in the cohort of women treated with multiple daily injections, lower CWB measured through the total scale of DQOL was associated with a higher level of HbA1c (*R* ^2^ = 0.55, *p* = .01).
Alvarado‐Martel et al^35^ Spain	100 patients with T1D. Mean age = 31.4 ± 11.6 years. Female, *n* = 45 (45%)	Es‐DQoL	HbA1c	Lower CWB measured through the total scale (*r* = 0.29, *p* < .001) and all subscales of Es‐DQoL (except for socio‐vocational concerns) was related to a higher level of HbA1c. Satisfaction subscale (*r* = 0.30, *p* < .001) Impact subscale (*r* = 0.30, *p* < .001) Worries subscale (*r* = 0.23, *p* < .05)
Santos et al^36^ Brazil	50 patients with T1D. Mean age 36.8 ± 11.3 years. Female, *n* = 25 (50%)	DQOL	HbA1c	CWB was not associated with HbA1c levels.
Castellano‐Guerrero et al^37^ Spain	312 patients with T1D. Mean age = 38.2 ± 12.7 years. Female, *n* = 45 (48.4%)	Es‐DQoL	HbA1c	HbA1c was not a predictor of CWB.
Thomakos et al^38^ Greece	80 patients with T1D 35.9 ± 11.4 years. Mean age 35.9 ± 11.4 years. Female, *n* = 43 (53.75%)	EuroQol: EQ‐5D EQ‐VAS	HbA1c	Higher CWB measured through EQ‐5D and EQ‐VAS was associated with a lower HbA1c level (*r* = −0.048, *p* = .036).
Benioudakis et al^39^ Greece	100 patients with T1D. Mean age 38.5 ± 13.9 years. Female, *n* = 81 (73.6%)	DQoL‐BCI	HbA1c	Higher CWB measured through the total score of DQoL‐BCI (95% CI) 3.84 (0.8, 6.9) *p* < .05 and the satisfaction with the treatment scale was related to better HbA1c (95% CI) 2.63 (0.8, 4.*4*) *p* < .05.
Anderson et al^40^ New Zealand	5887 patients with T1D. Three predetermined age groups: 8–12, 13–18, and 19–25 years. 19–25 years *n* = 1326. Female *n* = 649 (49%)	PedsQL Diabetes Module 3.0	HbA1c	Higher CWB measured through the total score of PedsQL was associated with a lower level of HbA1c in all three age groups. (95% CI) −3.2 (−4.0, −2.4) *p* < .001.
Bott et al^41^ Germany	657 patients with T1D. Mean age = 36 ± 11 years. Female, *n* = 42 (6.39%)	DSQOLS	HbA1c	Higher CWB measured through the subscales of physical complaints (*r* = −0.24*), concerns about the future (*r* = −0.17*), and satisfaction with treatment (*r* = −0.22*) was associated with a lower HbA1c level (*p < .001).
Wilmot et al^42^ India, Japan, Thailand, Bulgaria, Croatia, Serbia, Ukraine, Argentina, Brazil, Chile, Colombia, Iran, Saudi Arabia, France, Germany, Italy, and the UK	Patient‐reported outcomes in adults with T1D in global real‐world clinical practice: The SAGE study 1724 (44.7%) were aged 26 to 44 years, 1512 (39.2%) were aged 45 to 64 years, and 622 (16.1%) were aged 65 years or above. Mean age: 47.4 ± 14	ADDQoL	HbA1c	Good glycemic control for HbA1c (<7%) was associated with a greater CWB through Item 1 (present quality of life) (95% CI) 1.13 (1.06, 1.21) *p* < .001 and the total scores of ADDQoL (95% CI) 1.05 (1.01, 1.10) *p* = .020.
Tabaei et al^43^ USA	1522 patients (634 with T1D) mean age 33 (18–78). Female *n* = 342 (54%)	QWB‐SA	HbA1c	HbA1c was not significantly associated with CWB.

Abbreviations: ADDQoL, Audit of Diabetes‐Dependent Quality of Life; CI, confidence interval; CWB, cognitive well‐being; DQOL, Diabetes Quality of Life; DQOL‐BCI, DQOL brief clinical inventory; DSQOLS, Diabetes‐Specific Quality of Life Scale; EQ‐5D, EuroQuality of Life‐5D; EQ‐VAS, EQ‐visual analog scale; Es‐DQOL, Diabetes Quality of Life Spanish version; HbA1C, glycosylated hemoglobin; PedsQL, Pediatric Quality of Life inventory; QWB‐SA, Quality of Well‐Being Self‐Administered; T1D, type 1 diabetes.

**TABLE 2:** Prospective cohort studies analyzing the relationship between CWB and HbA1c

Study	Sample	Instruments	Outcome measures	Results
Hanna et al^44^ USA	T1: 184 emerging adults with T1D. Mean age = 18.2 ± 0.44 years. Female, *n* = 104 (56.5%). T2: 161 emerging adults (88%)	DQOL‐Y	HbA1c	HbA1c was not independently associated with any aspect of CWB.
Peasgood et al^45^ UK	2469 adults. Mean age: 39.3 ± 13.8 years. Female *n* = 1199 (48.6%)	EuroQol: EQ‐5D EQ‐VAS	HbA1c	Higher CWB measured through EQ‐5D and EQ‐VAS was associated with a lower HbA1c level. Random‐effects models, coefficient (SE). EQ‐5D: −0.0161 (0.003) *p* < .01 EQ‐VAS: −0.0164 (0.002) *p* < .01
Stahl‐Pehe et al^46^	560 patients. Female, *n* = 275 (49.1%). Subgroup age 11–13: 14–17: 18–21 (*n* = 114. 20.4%)	DISABKIDS diabetes module (DM)	HbA1c	Higher CWB measured through two subscales of DM (impact, *R* ^2^ = 0.58, *p* < .001 and treatment *R* ^2^ = 0.35, *p* < .001) was associated with a lower level of HbA1c at times 1 and 2 (Impact, *R* ^2^ = 0.116, *p* < .001 and treatment *R* ^2^ = 0.104, *p* < .001). However, this latter association disappeared when controlling for the HbA1c at time 1. Finally, the associations remained significant at time 2 for the impact subscale in patients with a deficient HbA1C at time 1 (*R* ^2^ = 0.407, *p* = .004)
Cooke et al^47^ USA	262 patients with T1D. Mean age 40 ± 14 years. Female, *n* = 131 (50%)	DSQOLS	HbA1c	HbA1c was negatively associated with CWB in bivariate analyses at baseline (*r* = −0.013, *p* < .05) but not in the following measures over time (post course), which was confirmed by multivariate analyses.

Abbreviations: CWB, cognitive well‐being; DQOL‐Y, Diabetes‐related Quality‐of‐Life Measure for Youths; DSQOLS, Diabetes Specific Quality of Life Scale; EQ‐5D, EuroQuality of Life‐5D; EQ‐VAS, EQ‐visual analog scale; HbA1C, glycosylated hemoglobin; T1D, type 1 diabetes; T2, type 2 diabetes.

Five of the 11 cross‐sectional studies explored the association between HbA1c and the DQOL questionnaire scores. First, Weinger and Jacobson^33^ measured CWB and HbA1c before and after an intensive diabetes treatment. They found a negative relationship between the satisfaction subscale of CWB and the HbA1c at baseline. This negative relationship was also achieved between changes in the same CWB subscale and those in HbA1c through the intervention. Thus, higher levels of satisfaction were related to lower HbA1c.

Second, Reddy et al^34^ divided their sample into two groups of patients according to the type of treatment: those who used insulin or continuous subcutaneous insulin infusion and those who used multiple daily injections (MDI). They found no correlation between HbA1c and the total score of CWB in the general study population. Nonetheless, in the subanalysis by gender, they found a positive association between HbA1c and the total score of CWB in the cohort of women who had MDI; that is, the higher the DQOL score (worst possible CWB), the higher the HbA1c levels.

Third, Alvarado‐Martel et al^35^ found that the most recent HbA1c measured in the participants was positively related to the total CWB score and all subscales except for socio‐vocational concerns. In other words, again, poor CWB was related to a higher HbA1c level. Specifically, multiple regression analyses showed how higher levels of HbA1c, being female, and the severity of the complications explained 25.2% of the total variance of the CWB score. Finally, two studies using the same questionnaire did not find any relationship between the two target variables.^36^, ^37^

On the other hand, Thomakos et al^38^ using the EuroQoL EQ‐5D, found a negative correlation between CWB and the HbA1c. Benioudakis et al,^39^ using the Diabetes Quality of Life brief clinical inventory (DQoL‐BCI), found that the CWB total score and the treatment satisfaction subscale are negatively associated with HbA1c. In the same direction, Anderson et al,^40^ using the Pediatric Quality of Life (PedsQL) Diabetes Module 3.0, also found a negative association between the CWB total score and the HbA1c. Overall, lower HbA1c was related to better CWB.

Bott et al,^41^ using Diabetes‐Specific Quality of Life Scale (DSQOLS), showed that three subscales (physical complaints, worries about the future, and treatment satisfaction) were negatively associated with HbA1c. Moreover, Wilmot et al,^42^ using the Audit of Diabetes‐Dependent Quality of Life (ADDQoL), found that an HbA1c <7% was associated with higher scores on the total and current CWB. Finally, Tabaei et al,^43^ using the Quality of Well‐Being Self‐Administered (QWB‐SA), did not find any association between CWB and HbA1C.

Regarding the longitudinal studies, Stahl‐Pehe et al,^46^ measured CWB twice (at baseline and after 3 years) using the DM questionnaire. They found a negative correlation between CWB and HbA1c at times 1 and 2. However, this latter association disappeared when controlling for the HbA1c at time 1. Finally, the correlations remained significant at time 2 for the impact subscale in patients with a deficient HbA1C at time 1. Moreover, Peasgood et al,^45^ using the EuroQoL EQ‐5D, found a negative correlation between CWB and the HbA1c. This relationship was found for the two parts of the questionnaire: the EQ‐VAS (EQ‐visual analog scale) and the EQ‐5D.

Cooke et al,^47^ using an adaptation of the DSQOLS, analyzed predictive factors influencing HbA1c and quality of life 1 year after structured education in flexible, intensive insulin therapy. Significant negative correlations were found only between the questionnaire and HbA1c in bivariate analyses at baseline (before the course). Nonetheless, these results were confirmed in multivariate analyses for none of the other measures over time (post course).

Finally, the study by Hanna et al,^44^ did not find any significant correlations between the variables studied.

In summary, of the 15 articles analyzed, 11 found associations between CWB and HbA1c, three of which used a large sample of patients.^40^, ^42^, ^45^ Four articles did not find any association between the key variables.

### 3.2 AWB and HbA1c

We found only one article that relates both variables (see Table 3), measuring AWB with the well‐being questionnaire. In this article, Eiser et al,^48^ found only a marginally significant correlation was found between the general AWB score and the HbA1c.

**TABLE 3:** A cross‐sectional study analyzing the relationship between AWB and HbA1c

Study	Sample	Instruments	Outcome measures	Results
Eiser et al^48^ UK	97 patients with T1D. Mean age = 47.81 ± 16.67 years. Female, *n* = 49 (50.51%)	Well‐being Questionnaire	HbA1c	Higher levels of HbA1c were related to marginally lower levels of general AWB (*r = −*0.20, *p* < .07).

Abbreviations: AWB, affective well‐being; HbA1C, glycosylated hemoglobin; T1D, type 1 diabetes.

In summary, the results obtained in this section do not allow us to draw conclusions or generalize, given the scarce literature. Nonetheless, as a starting point, it appears that lower levels of AWB are marginally and negatively associated with HbA1c levels in T1D.

## 4 DISCUSSION

The present systematic review has focused on analyzing how the two SWB factors (AWB and CWB) are related to HbA1c in adults with T1D. We hypothesized that higher levels of CWB and AWB will be negatively related to diabetes control, as indicated by better (lower) HbA1c levels. Therefore, we divided the results into two main sections: those comparing CWB with HbA1c (15 studies, of which 11 were cross‐sectional and 4 longitudinal) and those comparing AWB with HbA1c (1 cross‐sectional study).

In the first section, we included those studies in which the relationship between CWB and HbA1c was analyzed. A total of 11 of the selected 15 articles found significant associations between the two variables in the direction of the hypothesis proposed. Lower HbA1c was related to a higher level of CWB. Although one of the studies found the expected results only in a subsample of the participants,^34^ the overall results of these studies are consistent with our expectations considering the previous positive outcomes related to CWB found in the literature (eg, social support and self‐care behaviors).^27^, ^28^ Furthermore, in the persons with diabetes population, it has been found that CWB intervention programs have been associated with better medical outcomes.^49^ The protective component of CWB has also been studied in other medical conditions.^50^, ^51^

Interestingly, three of the studies analyzed showed gender differences in the relationship between HbA1c and CWB, as it appears to be stronger in women than in men.^34^, ^35^, ^40^ This result is supported by previous literature that has shown gender differences in the CWB variable, where women obtain worse scores than men.^52^, ^53^

On the other hand, four studies did not find any relationship between the target variables. These results were unexpected, given the previous literature. Various alternatives could explain this absence of significant effects. For example, Reddy et al,^34^ and Santos et al^36^ refer to the small number of evaluated T1D individuals. Other studies explain that the unexpected results may be because of the use of well‐controlled diabetes participants who could not be entirely representative of the general T1D.^34^, ^43^ The study by Hanna et al^44^ relied on an emerging adult sample that, although with poor diabetes control, showed limited variability in their HbA1c, which may have reduced the possibility of detecting an association between the target variables.

In the second section, we included those studies in which the relationship between AWB and HbA1c was analyzed. However, we found only one study. Eiser et al,^48^ found that those participants with higher levels of HbA1c showed lower levels of global AWB, although this relationship was only marginally significant. These results are inconclusive, given the insufficient number of studies. Thus, future studies should focus on extending this promising line of research. Previous studies with T1D adolescents have confirmed our hypothesis regarding AWB and HbA1c; that is, greater positive affect appeared to be related to better HbA1c.^54^, ^55^

The present systematic review suggests the potential value of considering CWB as a critical component for good HbA1c. However, the studies of the current review are not exempt from limitations. First, some of the studies use a sample with an extensive age range,^42^, ^43^ which can affect the relationships found because HbA1c and CWB can differ throughout various life stages. Second, nine different CWB questionnaires have been employed across the studies analyzed. This variability makes it difficult to group the results according to the type of measure and allows us to present only descriptive results, despite the meta‐analysis carried out.

Moreover, all except two questionnaires were specific to diabetes, and the others were related to health perception. CWB is a more extensive concept that could include a more general perspective on life. Third, we have found only one article regarding AWB, making it difficult to draw any generalizable conclusions and suggesting that more research is needed on this issue. Fourth, the sample used in this review includes only adults. Therefore, future investigations should attempt to evaluate the impact of SWB interventions on glycemic control in adolescents because the literature has shown that HbA1c levels are worse at puberty.^56^, ^57^ Finally, the studies included in the review use correlations and cannot predict causality. Thus, the present review does not allow us to predict if the poor glycemic control causes the decrease in SWB or vice versa.

This systematic review helps us better understand the relationship between SWB (through the CWB variable) and HbA1c in adults with T1D. The overall data suggest that CWB is negatively related to HbA1c in this population, but these results are inconclusive. It is therefore necessary to continue investigating other variables that may influence the well‐being of people with T1D.

This finding could have several clinical implications, such as the possible evaluation, prevention, and treatment of the problems associated with diabetes through the study and training of the psychosocial variables that may directly influence SWB.

## CONFLICT OF INTEREST

The authors declare that the research was conducted without any commercial or financial relationships that could be construed as a potential conflict of interest.

## Data Availability

Data sharing is not applicable – no new data generated.

## APPENDIX A

**TABLE A1:** Mixed methods appraisal tool (MMAT)

Quantitative non‐randomized studies	(Weinger & Jacobson^33^)	(Reddy et al,^34^)	(Santos et al,^36^)	(Castellano‐Guerrero et al,^37^)	(Hanna et al,^44^)	(Thomakos et al^38^)	(Peasgood et al,^45^)	(Benioudakis et al,^39^)	(Anderson et al,^40^)	(Stahl‐Pehe et al,^46^)	(Wilmot et al^42^)	(Bott et al, 1998)	(Tabaei et al, 2004)	(Cooke et al, 2015)	(Eiser et al, 2007)
S1. Are there clear research questions?	Yes	Yes	Yes	Yes	Yes	Yes	Yes	Yes	Yes	Yes	Yes	Yes	Yes	Yes	Yes
S2. Do the collected data allow to address the research questions?	Yes	Yes	Yes	Yes	Yes	Yes	Yes	Yes	Yes	Yes	Yes	Yes	Yes	Yes	Yes
1.1. Are the participants representative of the target population?	Yes	Yes	Yes	Yes	Yes	Can't tell	Yes	Yes	Yes	Yes	Yes	Yes	Yes	Yes	Yes
1.2. Are measurements appropriate regarding outcome and intervention (or exposure)?	Yes	No	Yes	Yes	Yes	Yes	Yes	Yes	Yes	Yes	Yes	yes	Yes	Yes	Yes
3.3. Are there complete outcome data?	Yes	Yes	Yes	No	Yes	Yes	Yes	Yes	Yes	No	Yes	Yes	Yes	Yes	No
1.4. Are the confounders accounted for in the design and analysis?	Can't tell	Yes	Yes	Yes	Yes	Yes	Yes	Yes	Yes	Yes	Yes	Can't tell	Yes	Yes	Yes
1.5. During the study period, is the intervention administered (or exposure occurred) as intended?	Yes	Yes	Yes	Yes	Yes	Yes	Yes	Yes	Yes	Yes	Yes	Yes	Yes	Yes	Yes
Mixed methods	Alvarado‐Martel et al^35^														
S1. Are there clear research questions?	Yes														
S2. Do the collected data allow to address the research questions?	Yes														
2.1. Is there an adequate rationale for using a mixed‐methods design to address the research question?	Yes														
2.2. Are the different components of the study effectively integrated to answer the research question?	Yes														
2.3. Are the outputs of integrating qualitative and quantitative components adequately interpreted?	Yes														
2.4. Are divergences and inconsistencies between quantitative and qualitative results adequately addressed?	No														
2.5 Do the different components of the study adhere to the quality criteria of each tradition of the methods involved?	Yes														

## APPENDIX B Instruments

We next define the instruments used to measure cognitive well‐being (CWB) and affective well‐being (AWB) in the studies selected. Those subscales that measure anxiety or depression in both the cognitive and affective components of well‐being will not be taken into account, because our objective is to evaluate subjective well‐being from a positive psychology approach.^58^ Many studies have linked this disease with negative factors, and from this perspective we wish to evaluate the factors that positively influence or improve the well‐being of these people.

### CWB Instruments

- The Diabetes Quality of Life (DQOL).^59^ This questionnaire consists of 46 items and provides a total score of general well‐being. In addition, it is divided into the four subscales of satisfaction, impact, diabetes‐related worries, and worries about social and vocational aspects. A higher score indicates a lower CWB. Cronbach's alpha values for the internal consistency of the four subscales ranged between 0.66 and 0.92, with a test–retest reliability *r* = 0.78–0.92.
- Diabetes‐related Quality‐of‐Life Measure for Youths (DQOL‐Y).^60^ This questionnaire is composed of three intercorrelated subscales: A Disease Impact Scale (23 items), a Disease‐Related Worries Scale (11 items), and a Diabetes Life Satisfaction Scale (17 items), plus one single item on health perception. Each item is scored on a scale ranging from 1 (never or very dissatisfied) to 5 (all the time or very satisfied). For the 1‐item rating of health, participants were asked to rate their health as poor (1), fair (2), good (3), or excellent (4). Lower scores indicated a poorer CWB. Reliability for each of the three subscales, life satisfaction, the impact of diabetes, and worries about diabetes were denoted by alpha values of 0.90, 0.85, and 0.84, respectively.^44^
- EuroQoL EQ‐5D.^61^ This is a generic measure of health‐related quality of life, divided into two parts: the EQ‐5D and the EQ‐VAS (visual analog scale). The EQ‐5D comprises five subscales: mobility, self‐care, habitual activities, pain/discomfort, and anxiety/depression. Each question is answered on a scale from 1 (no problem) to 3 (severe problem). The EQ‐VAS is used to assess the current CWB of the respondent. The score ranges from 0 (worst possible health) to 10 (best imaginable health). The reliability for EQ‐VAS and EQ‐5D was *α* = 0.78 and *α* = 0.73, respectively.
- Diabetes Quality of Life‐Brief Clinical Inventory (DQoL‐BCI).^62^ This consists of 15 questions divided into two subscales: negative impact and satisfaction with treatment. This questionnaire estimates patient satisfaction, the level of disease monitoring, and self‐care attitudes. Responses are rated on a 5‐point Likert scale, ranging from 1 (very satisfied/never) to 5 (very dissatisfied/constantly). This scale offers a total score ranging from 15 to 75, where higher scores reflect poorer CWB.^63^ The internal consistency of the questionnaire was shown by *α* = 0.95.^62^
- The Pediatric Quality of Life Inventory 4.0 (PedsQLTM type 1 diabetes module).^64^ This is a quality‐of‐life measure that evaluates young people's perceptions of their health. The questionnaire consists of 28 items, divided into five subscales: diabetes symptoms (11 items), treatment barriers (four items), treatment adherence (seven items), worry (three items), and communication problems (three3 items). In addition, it offers a total score that ranges from 0 to 100. Therefore, only the total score was used in our review. A higher score denotes a better CWB. The mean Internal consistency for the type 1 diabetes module was *α* = 0.71.
- DISABKIDS Diabetes Module (DM).^65^ In this scale, CWB is measured through two subscales: the impact scale (six6 items) and the treatment scale (four4 items). The score of both scales ranges from 0 to 100, with higher scores indicating a better CWB. The internal consistency for the questionnaire was *α* = 0.76 for the impact scale and *α* = 0.84 for the treatment scale.^46^
- The Diabetes‐Specific Quality‐of‐Life Scale (DSQOLS).^41^ This questionnaire specifically assesses the four main components of CWB in patients with type 1 diabetes (physical, emotional, and social burdens together with daily functioning). The instrument consists of 64 items, and responses are scored on a six‐point Likert scale. The questionnaire is divided into the following subscales: treatment goals (10 items); treatment satisfaction according to treatment goals (10 items); physical complaints (10 items); emotional burdens and concerns (8 items); social problems (9 items); daily functions (work, leisure, time requirements; 11 items); and dietary restrictions (6 items). Factor analysis revealed six reliable components (Cronbach's *α*): social relations (0.88), worries about future (0.84), physical complaints (0.84), diet restrictions (0.71), leisure time flexibility (0.85), and daily hassles (0.70).
- Audit of Diabetes‐Dependent Quality of Life (ADDQoL) questionnaire. This questionnaire consists of 19 items that measure the impact of the disease in different areas of life and how these affect their CWB. In addition, two other general elements measure current CWB (Item 1) and what CWB would be like without diabetes (Item 2). Higher scores on Item 1 indicate a higher CWB, and lower scores on Item 2 indicate better potential CWB without diabetes. The internal consistency of the questionnaire was *α* = 0.92.^66^
- The Quality of Well‐Being Self‐Administered (QWB‐SA)^67^ consists of 71 items that assess symptoms and disease functioning across three domains: mobility, physical activities, and social activities of symptoms/problems, to provide a score as a total measure of CWB. This scale allows the placement of each individual on a continuum of well‐being ranging from 0 (zero well‐being) to 1.0 (asymptomatic and fully functional). The test–retest reliability was *r* = 0, 77.

### AWB Instruments

Well‐being Questionnaire^68^: This questionnaire consists of 22 items that are divided into four subscales: depression (six6 items), anxiety (six items), energy (four4 items), and positive well‐being (six6 items). Each item is evaluated on a scale ranging from 0 to 3. A higher score indicates greater general AWB. Reliability for each of the four subscales was depression, *α* = 0.74, anxiety, *α* = 0.78, energy, *α* = 0.77 and positive well‐being, *α* = 0.86.^48^
